# Neutrophil Elastase and Chronic Lung Disease

**DOI:** 10.3390/biom11081065

**Published:** 2021-07-21

**Authors:** Judith A. Voynow, Meagan Shinbashi

**Affiliations:** 1 Division of Pediatric Pulmonology, Children’s Hospital of Richmond at Virginia Commonwealth University, Richmond, VA 23298, USA; 2 School of Medicine, Virginia Commonwealth University, Richmond, VA 23298, USA; shinbashim@mymail.vcu.edu

**Keywords:** neutrophil elastase, cystic fibrosis, chronic obstructive pulmonary disease, bronchiectasis, bronchopulmonary dysplasia, antiprotease, glycosaminoglycan

## Abstract

Neutrophil elastase (NE) is a major inflammatory protease released by neutrophils and is present in the airways of patients with cystic fibrosis (CF), chronic obstructive pulmonary disease, non-CF bronchiectasis, and bronchopulmonary dysplasia. Although NE facilitates leukocyte transmigration to the site of infection and is required for clearance of Gram-negative bacteria, it also activates inflammation when released into the airway milieu in chronic inflammatory airway diseases. NE exposure induces airway remodeling with increased mucin expression and secretion and impaired ciliary motility. NE interrupts epithelial repair by promoting cellular apoptosis and senescence and it activates inflammation directly by increasing cytokine expression and release, and indirectly by triggering extracellular trap release and exosome release, which magnify protease activity and inflammation in the airway. NE inhibits innate immune function by digesting opsonins and opsonin receptors, degrading innate immune proteins such as lactoferrin, and inhibiting macrophage phagocytosis. Importantly, NE-directed therapies have not yet been effective in preventing the pathologic sequelae of NE exposure, but new therapies are being developed that offer both direct antiprotease activity and multifunctional anti-inflammatory properties.

## 1. Introduction

Neutrophil elastase (NE) is a major proteinase in primary granules in neutrophils that participates in microbicidal activity [1,2,3]. NE (Leukocyte elastase, EC 3.4.21.37), a serine endopeptidase, is characterized by serine in the active tripeptide catalytic site: Asp, His, Ser [4]. NE is the most abundant of four serine proteases present in neutrophils, which also include proteinase 3, cathepsin G, and neutrophil serine protease 4 (NSP4) [5]. NE is a 29.5 kD protein stored in mature form in the azurophilic granules of neutrophils and is present at high concentrations per azurophilic granule—approximately 67,000 molecules (~5 mM) per granule. NE localization to azurophilic or primary granules requires serglycin, a proteoglycan with chondroitin sulfate residues, which bind to the basic amino acid residues of NE [6]. NE is also localized to the cell surface after neutrophil activation by binding to highly abundant but low-affinity chondroitin sulfate and heparan sulfate proteoglycans [7]. NE activity at the neutrophil surface occurs in quantum proteolytic bursts attributed to the high local concentration of NE, which overwhelms local antiprotease concentrations for the initial seconds of activity [8]. NE cleaves neutral, non-aromatic dipeptides, and thus, has a broad array of substrates (reviewed in [9,10]). Several factors modulate NE airway protease activity, including the abundance and localization of NE, the concentrations of lung antiproteases, α_1_-antitrypsin, α_2_-macroglobulin, or secretory leukocyte proteinase inhibitor (SLPI), at the site of enzyme activity [8], and even the target protein O-linked glycosylation [11]. Although NE proteinase activity is critical for normal innate immune function, release of NE into the airway milieu contributes to lung disease progression. In this review, we will discuss the pathogenesis of dysregulated NE release into the airways in chronic lung diseases, and the mechanisms by which NE alters airway and lung parenchymal structures, and modulates innate immune processes and inflammation, leading to worsening disease. We will review the status of clinical trials to target NE in chronic lung diseases, and alternative strategies to control NE-mediated lung injury.

## 2. Neutrophil Elastase Is Required for Microbial Clearance

Exposure of neutrophils to cytokines, chemokines, or bacterial products activates granule fusion with the plasma membrane and NE localization at the cell surface for chemotaxis to the site of infection. During neutrophil phagocytosis, NE plays a critical role in microbial killing by fusing primary granules with phagolysosomes and releasing NE, which digests the microorganisms in concert with microbicidal peptides and reactive oxygen species (ROS) generated by NADPH oxidase and myeloperoxidase [4]. NE antimicrobial activity is required for clearance of Gram-negative bacteria [2]. Specifically, NE cleaves the *E. coli* outer membrane protein A (OMP A), resulting in bacterial death. NE-knockout mice exposed to *E. coli* have greater mortality than wild-type mice, with uncontrolled infection and death [1]. NE also participates in extracellular microbial killing by neutrophil extracellular traps (NETs). NETs are extracellular DNA webs released by neutrophils. They are composed of DNA-adherent pro-inflammatory proteins including NE, myeloperoxidase, histones and High Mobility Group Box 1, that together with DNA, adhere to and destroy microbes [12].

However, when NE extracellular release is dysregulated, NE can remodel the airways and lung parenchyma, promote sustained inflammation, and impair the innate immune system; together, these actions contribute to lung disease pathogenesis (summarized in Table 1).

## 3. NE-Dependent Mechanisms Inducing Airway Mucus Obstruction

The major macromolecular component of airway secretions is airway mucins (for a detailed review, see reference [49]). Mucins are large polymeric glycoproteins. Some mucins are cell-associated and are required to maintain airway hydration homeostasis for normal ciliary motility [50], while secreted gel-forming mucins, released from submucosal glands and goblet cells, are critical for clearing microbes and pollutants from the airway via the mucociliary escalator. However, in chronic lung diseases, gel-forming mucin expression is upregulated by viruses, bacteria, pollutants, and neutrophil mediators including reactive oxygen species (ROS) and NE, resulting in hyperconcentrated airway mucus, a condition associated with mucostasis and airway mucus obstruction [51].

NE upregulates the gene expression of one of the major gel-forming, secreted mucins in the airway, *MUC*5AC, by both transcriptional and post-transcriptional mechanisms [13]. *MUC*5AC expression is upregulated by intracellular signals activated by NE-including ROS [52,53,54], NADPH quinone oxidoreductase 1 (NQO1) [53] and epidermal growth factor receptor (EGFR) activation [55]. NE facilitates transforming growth factor α (TGFα) -induced EGFR ligation by a coordinated mechanism: 1) releasing cell surface TGFα, and 2) increasing epithelial permeability by degrading Zona occludins-1 [56] and E-cadherin [57] junctional proteins, thereby permitting TGFα, a basolateral ligand, access to activate EGFR, an apical receptor [58]. In addition to upregulation of MUC5AC, NE also activates secretion of mucins from primary human bronchial epithelial cells [59], which contributes to mucus obstruction of airways. Finally, NE increases goblet cell metaplasia, which alters the epithelial composition in the airway and perpetuates increased mucin production and secretion. NE induces goblet cell metaplasia in a murine model [21] via Nqo1 [60] and tumor necrosis factor (TNF) alpha converting enzyme (TACE) [61]. In addition to NE, goblet cell metaplasia is increased by cytokines including IL-13, IL-1β, IL-6, IL-17, IL-9, TNF-α, and increased by microbes and microbial products, including lipopolysaccharide (LPS), *M. pneumoniae, P. aeruginosa* [62], rhinovirus [63] and non-typeable *H. influenza* [63]. The presence of cigarette smoke [63] or ozone [64] further enhances goblet cell hyperplasia (reviewed in [49,65,66]).

To generate effective mucociliary clearance requires normal mucin abundance, sufficient airway surface liquid hydration, and a healthy ciliated epithelium. NE interferes with each of these components required for normal mucociliary clearance. In addition to increasing mucin abundance in the airway, NE impairs airway surface liquid hydration by degrading cystic fibrosis transmembrane conductance regulator (CFTR) [30], an apical chloride channel, and activating ENaC [31], an apical epithelial sodium channel, resulting in perturbed ionic regulation of airway hydration. In addition, NE decreases ciliary motility and injures ciliary structures [24,67]. The failure of ciliary motility in concert with airway surface liquid dehydration and increased mucin production results in mucus obstruction of airways, a hallmark of chronic inflammatory airway diseases such as cystic fibrosis (CF), chronic obstructive pulmonary disease (COPD), and asthma.

## 4. NE Alters Cellular Differentiation and Cellular Fate

In addition to degrading mucociliary clearance and contributing to airway mucus obstruction, NE protease activity degrades extracellular matrix (ECM) proteins, particularly elastin, which is critical for alveolar structure [68,69]. NE-mediated ECM degradation causes direct pulmonary alveolar injury, resulting in emphysema, and subepithelial fibrosis [70] following the release of the pro-fibrotic factor, transforming growth factor β1 (TGF-β1), from the ECM [71,72]. Thus, NE activity contributes to the progression of COPD with emphysema, and CF and asthma with increased basement membrane thickening.

NE injures epithelial cells and the severity of the insult influences epithelial cell fate. After NE exposure and loss of epithelial cells, DNA synthesis is transiently inhibited and this is associated with degradation of ErbB2, an EGFR receptor [38]. However, NE also upregulates MUC4 [14], a ligand for ErbB2, and activation of ErbB2 increases epithelial proliferation and differentiation [38].

NE exposure activates at least three different epithelial cell fates. NE upregulates the cyclin kinase inhibitor p27 kip1, which interrupts cell cycle progression, causing cell cycle arrest, and a state called quiescence, which is reversible [41]. NE induces epithelial apoptosis via protease-activated receptor 1 (PAR1) activation [73], triggering phagocytosis by macrophages, which detect the apoptotic cell surface marker, phosphatidyl serine, to clear these cells, a process called efferocytosis. However, in the lungs of patients with CF or bronchiectasis, high concentrations of NE also cleave the macrophage phosphatidylserine receptor, resulting in efferocytosis failure and increased burden of apoptotic cells that undergo necrosis in the lung [23]. NE also activates senescence markers in airway epithelia [42], which are present at greater levels in CF airway tissue than in non-CF airway tissue. Cellular senescence also occurs in COPD and may be activated by oxidative stress and DNA injury [74]. Senescence due to cellular stress is associated with three genetic indicators: (1) upregulation of p16 ^INK4a^, a cyclin-dependent kinase 4 inhibitor, (2) markers of DNA injury phospho-histone 2AX (γ-H2A.X) and phospho-checkpoint kinase 2 (p-Chk2) and (3) telomerase shortening [42]. Importantly, senescent cells activate NF-κB, resulting in the senescence-associated secretory phenotype (SASP) [75]. The SASP is characterized by the release of proinflammatory cytokines, IL1β, IL-6, TNF-α, chemokines, CXCL1, CXCL8, CCL2, matrix metalloproteinases, MMP-2, MMP-9, and growth factors, TGF-β [75]. Thus, NE propagates a vicious cycle of pro-inflammatory signaling and accumulation of pro-inflammatory senescent cells in CF and COPD [75].

## 5. NE Activates Pro-Inflammatory Signaling

NE upregulates epithelial expression of CXCL8 (IL-8), a major neutrophil chemokine, via TLR4 signaling, which activates downstream signals MyD88, IL-1 receptor-activated kinase (IRAK), and tumor necrosis factor receptor-activated factor-6 (TRAF-6), leading to NFκB translocation into the nucleus [76]. NE also upregulates CXCL8 via activation of meprin α, a metalloprotease, that activates EGFR by releasing the EGFR ligand TGFα [16]. Thus, NE activates both TLR4 and EGFR to increase CXCL8 gene regulation and protein production. In addition to CXCL8, extracellular NE activates IL-1α, IL-33 and IL-36α and γ [22] from epithelial cells, and TNFα and IL-1β from macrophages [37], resulting in increased neutrophil chemotaxis and airway inflammation. NE activates the release of High Mobility Group Box 1 (HMGB1), a damage-associated molecular pattern, from macrophages [25], which is associated with more severe lung disease in CF [77,78] and COPD [79]. NE protease activity also generates fibrin degradation products that are chemotactic for neutrophils [48]. By all these mechanisms, NE proteolytic activity creates airway signals that perpetuate neutrophilic inflammation.

NE also increases airway inflammation by other mechanisms. NE increases the abundance of a pro-inflammatory sphingolipid, long chain ceramide, in murine airways by upregulating serine palmitoyl transferase long chain subunit 2 (SPTLC2), the enzyme that catalyzes the rate-limiting step for ceramide generation. Inhibition of SPTLC2 prevents NE upregulation of ceramides and blocks the release of an inflammatory cytokine, keratinocyte-derived chemokine (KC, the murine analogue of CXCL8), and HMGB1 [36]. In CF sputum, NE concentration correlates with increased ceramide abundance [80]. NE generates an outside-to-inside signal for oxidative stress by degrading heme-containing proteins in the extracellular space, resulting in heme-free iron, which is taken up by cells and creates intracellular oxidative stress [32]. NE is a master regulator of protease activation and antiprotease destruction in the airway (for review, see [81]). Free NE activates pro-MMP-9, which is associated with bronchiectasis in CF [82]. NE degrades tissue inhibitor of matrix metalloprotease-1 (TIMP-1), and degrades the anti-elastase inhibitor, secretory leukoprotease inhibitor [43], resulting in sustained protease activity.

NE stimulates the release of neutrophil extracellular traps (NETs) [46], DNA web-like structures that have attached chromatin and granule proteins including NE, myeloperoxidase, HMGB1 and antimicrobial proteins. NE activates NET release through a highly orchestrated signaling pathway, requiring myeloperoxidase and hydrogen peroxide for release from the azurophilic granule into the cytosol, association with F-actin during this transit, then degradation/release of actin for NE entry into the nucleus [83]. Once in the nucleus, NE clips Histone H4, which is required for chromatin decondensation and NET release [83]. Although NETs have been assigned as antimicrobial structures [12], they can also activate the inflammasome [84].

Neutrophil exosomes are nm-diameter vesicular structures released into the extracellular milieu that share membrane phospholipids derived from neutrophil endosomes and plasma membrane [47]. Exosomes carry cargo including both membrane and cytosolic proteins, lipids, metabolites, RNA and DNA [85]. Neutrophil exosomes are released spontaneously from cells and, under control circumstances, have no protease on the exosome surface [47]. However, activated neutrophil exosomes treated with FMLP or harvested from inflamed airways, including bronchoalveolar lavage fluid (BALF) of patients with COPD, harbor high concentrations of surface NE [47]. When instilled into mouse lungs, both activated neutrophil exosomes and COPD and bronchopulmonary dysplasia (BPD) BALF exosomes cleave ECM and are sufficient to cause emphysema [47]. Thus, neutrophil exosomes harbor high NE concentrations at the membrane surface, which are protected from airway antiprotease inhibition.

## 6. NE Impairs Innate Immunity

Although extracellular NE activity promotes inflammation, which should result in clearance of microbes, this antimicrobial function is thwarted when NE activity is dysregulated and overcomes the normal antiprotease capacity in the lung. The reason for this failure is that excessive NE interferes with innate and adaptive immune mechanisms. NE cleaves antimicrobial proteins, lactoferrin [86] and midkine [20], important for bacterial clearance. NE cleaves several opsonins including the surfactant protein (SP) collectins SP-D [28] and SP-A [29], complement C3bi and its receptor CR1 [26], and pseudomonas-specific IgG [27]. Altogether, the cleavage and loss of opsonins and phagocytic receptors due to NE activity [37] result in failure of both neutrophil and macrophage microbial killing and clearance. NE degrades monocyte CD14, a major monocyte receptor for bacterial lipopolysaccharide (LPS) [87]. NE also impairs maturation of monocyte-derived dendritic cells (mDC) by cleaving cell surface receptors, CD40, CD80, and CD86, resulting in failure of mDC to prime lymphocyte proliferation or cytokine production in response to antigen [88]. In CF airways, apoptotic neutrophils display the “eat-me” signal of increased plasma membrane phosphatidylserine, but these cells fail to be phagocytosed and cleared by macrophages due to NE-mediated degradation of the macrophage phosphatidylserine receptor [23]. Failure of apoptosis contributes to inflammation, as apoptotic cells undergo necrosis, releasing DNA and granular inflammatory mediators.

## 7. NE and Cystic Fibrosis Lung Disease

Although CF affects many organ systems, the major cause of morbidity and mortality is chronic lung disease due to infection and inflammation of the airways, which leads to bronchiectasis and respiratory failure [89]. Mutations in the CF Conductance Regulator (CFTR) gene are the primary defect in CF with loss of function of this anion channel. This defect causes abnormal mucus, which is adherent to airway epithelia [90,91], causing mucociliary clearance failure, and recurrent bronchitis. Although exacerbations of bronchitis lead to neutrophilic inflammation, the primary defect associated with airway mucus obstruction is sufficient to induce sustained neutrophilic inflammation, even in the absence of infection/presence of antibiotics [92]. There is strong evidence that NE participates in many of the pathogenic events that lead to chronic lung disease in CF. NE is present very early in the airway in CF infants in bronchoalveolar lavage fluid (BALF) and is associated with computer tomographic (CT) evidence of bronchiectasis in children with CF [93]. Over the first 6 years of life in children with CF, detection of bronchial lavage fluid NE correlates more closely with progressive structural lung damage (bronchiectasis and mucus obstruction of airways on CT) than infections [94]. The amount of free NE in sputum has also been shown to correlate inversely with FEV_1_ in children with CF [95].

NE impairs mucociliary function and innate immune function and increases inflammation in the CF lung by several mechanisms. NE upregulates the gel-forming mucin, MUC5AC. NE activates the apical epithelial sodium channel [31], which increases sodium uptake from the airway surface liquid (ASL) and contributes to ASL dehydration and airway mucus obstruction. NE upregulates epithelial expression of CXCL8 [18,96], a chemokine that increases NE release from CF neutrophils, resulting in a self-perpetuating cycle of neutrophil inflammation and overabundant NE in ASL [97]. In addition, NE degrades secretory leukoprotease inhibitor (SLPI) [43], activates the neutrophil metalloprotease gelatinase (MMP-9), and degrades tissue inhibitor of metalloprotease-1 (TIMP-1), further sustaining overwhelming proteolytic inflammation [40]. The abundance of airway NE is also associated with airway remodeling with increased airway basement membrane thickness [71], and premature epithelial senescence [42]. Excess NE impairs both the innate and adaptive immune systems by degrading antimicrobial proteins in the ASL, cleaving opsonins and opsonin receptors [98], and generating oxidative stress in airway epithelia [99]. Finally, NE as well as myeloperoxidase, bacteria, ROS and other stimuli activate release of NETs [46] that add DNA to the airway, increasing the viscoelasticity of airway mucus [100]. NETs contain cargo including NE and other pro-inflammatory proteins that contribute to persistent inflammation in the CF airway and the progression of lung disease [99].

## 8. NE and Chronic Obstructive Pulmonary Disease

COPD is the third leading cause of death globally [101]. It is commonly associated with cigarette smoking, exposure to biomass fuel combustion and air pollution, and is characterized by persistent inflammation and progressive airflow limitation. As with CF, neutrophilic inflammation is a notable feature of COPD. Exposure to irritants such as cigarette smoke and pollutants triggers the release of a cytokine network that promotes neutrophil recruitment, resulting in a protease–antiprotease imbalance [102] and establishing a vicious cycle of inflammation and airway remodeling [103]. Sputum neutrophil counts have been shown to correlate with the rate of lung function decline [104] and peripheral airway dysfunction [105]. Acute exacerbations of bronchitis due to bacterial or viral infections are the major cause of morbidity and mortality in COPD [106] and are associated with elevated NE levels [107]. NE and other proteases cooperate to regulate the protease–antiprotease activity in the COPD airway. For example, there is compelling evidence that MMP-12/Macrophage elastase is required for emphysema after smoke exposure [108]. NE activates MMPs and cysteinyl cathepsins that induce emphysema, and NE sustains MMP activity by degrading TIMP-1, a major inhibitor of MMPs [109]. Reactive oxygen species oxidize and inactivate α-1-antitrypsin, resulting in unrestrained NE activity [110]. NE and MPO activate the release of NETs into the airway milieu that propagate NE and neutrophil granule proteolytic and pro-inflammatory activities [47]. NET abundance in the COPD airway is associated with decreased lung function, increased exacerbations, and with diminished neutrophil phagocytosis in the COPD airway [111]. The effects of NE are especially prominent in α1- antitrypsin deficiency, where decreased amounts or complete loss of α1-antitrypsin result in the unopposed actions of NE and subsequent destruction of the alveolar matrix [112]. There is synergy between NE and MMP-12 (macrophage elastase) to promote tobacco smoke-induced COPD lung pathology, as MMP-12 degrades the NE inhibitor, α-1 antitrypsin [108] and NE degrades the MMP-12 inhibitor, TIMP1 [40], resulting in unrestrained protease activities.

## 9. NE and Bronchiectasis

Bronchiectasis is a disease that is defined by permanent and abnormal airway widening with mucus obstruction [51] and subsequent airflow obstruction [113]. Bronchiectasis may be due to inherited diseases such as primary ciliary dyskinesia or primary immunodeficiencies. However, bronchiectasis may also be caused by mechanical airway obstruction, recurrent insults such as aspiration, secondary immunodeficiency, or severe bacterial or viral pneumonia and subsequent airway injury [114]. Although the pathogenesis of non-CF bronchiectasis is not fully understood, there is increasing evidence that neutrophils are associated with its progression. For example, bronchiectatic airways have higher levels of neutrophil infiltration in the lamina propria compared to control airways [115]. One commonly proposed hypothesis for pathogenesis is that an initial bacterial infection of the lower respiratory tract triggers an exaggerated and uncontrolled neutrophilic airway inflammatory response [116]. This results in damaged airways with impaired mucociliary clearance and increased susceptibility to severely damaging pathogens such as *P. aeruginosa*, leading to further inflammation [117]. Another hypothesis for pathogenesis is that an insult causes the initial event of mucus obstruction of the airway sufficient to activate both macrophage and epithelial signaling and lead to neutrophil activation [51]. NE proteolytic activity has been shown to correlate with decreased lung function [118] and increased susceptibility to airway bacterial colonization [119], indicating NE as a potential biomarker of disease severity. Furthermore, sputum NE activity in patients with bronchiectasis is associated with an increased risk and frequency of exacerbations, hospitalizations, and mortality [120]. This is the rationale for a phase 2 study of AZD9668, an oral NE inhibitor, which was tested in patients with bronchiectasis in a randomized, double-blind, placebo-controlled trial over 4 weeks [121]. Although AZD9668 was not associated with adverse side effects, efficacy was modest, with only a small increase in FEV_1_ (100 mL) and no significant changes in sputum NE or IL-8 or patient symptoms survey results [121]. The lack of efficacy of AZD9668 may be due to the small number of subjects enrolled and subject variability. Therefore, it is still not clear whether targeting NE activity alone will be a successful therapeutic strategy for bronchiectasis. However, NE may still be a useful marker for the clinical assessment of patients with bronchiectasis and may also identify patients at the highest risk of disease progression. The potential that airway NE concentrations may serve as a biomarker for disease activity would be an important advance, given that there are currently no gold standards for measuring inflammation in bronchiectasis [122]. A point-of-care sputum NE activity assay is established [123]; however, future studies will be necessary to evaluate whether sputum NE measurements will translate into improved outcomes for bronchiectasis.

## 10. NE and Bronchopulmonary Dysplasia

Bronchopulmonary dysplasia (BPD) is a chronic lung disease that occurs in premature infants and is defined by the requirement for supplemental oxygen at 36 weeks post-gestational age [124,125]. BPD is attributed to an arrest in lung development and is the end result of a complex process where factors including gestational age, birth weight, ventilatory support, and oxygen toxicity compromise normal lung development [126]. This leads to a sustained reduction in lung function with airspace enlargement and altered capillary development. Inflammation is a key component in the pathogenesis of BPD, as chorioamnionitis and postnatal sepsis are associated with this disease [127,128]. There is also increasing evidence that NE is a key mediator in BPD, as NE is elevated in BPD airways [129] and has increased enzymatic activity on the surface of neutrophil exosomes obtained from tracheal aspirate of infants with BPD [47]. A recent study using a transgenic mouse model for NFκB activation in the airway found that sublethal inflammation from NE instillation during the saccular stage of lung development, but not during the alveolar stage of development, resulted in a BPD-like lung phenotype of enlarged simplified alveoli, while neutrophil-depleted mice showed normal alveolar structure [130]. NE was also found to be elevated in airways of the mice with lung disease, strongly suggesting that excess NE proteolytic activity leads to aberrant lung development. Culturing lung fibroblasts from these mice revealed that NE or neutrophil exosomes from tracheal aspirate of infants with BPD downregulate the mRNA expression of elastin assembly genes, further implicating the involvement of NE in the pathogenesis of BPD. Finally, the NE-exposed mice had aberrant lung structure that persisted into adulthood and resembled emphysema. Future studies should investigate the role of NE during this critical period and whether anti-NE therapies can reduce the risk of developing BPD and subsequent COPD in adult life.

## 11. NE Inhibitors and Mechanisms of Action

Given the increasing evidence for NE playing a major role in the pathogenesis of chronic lung diseases, there is a need for developing NE-targeted therapies. One potential strategy is to directly address the protease–antiprotease imbalance seen in these diseases by increasing antiprotease function. Replacement therapy has been approved for patients with α1-antitrypsin deficiency based on evidence of NE inhibition. In three large multicenter placebo-controlled, randomized, double-blind trials, α1-antitrypsin infusion stops progression of emphysema, as determined by CT scores over a 2-year time course [131,132,133]. However, there is no evidence that α1-antitrypsin replacement therapy affects risk for exacerbations or improves lung function in patients without genetic α1-antitrypsin deficiency. At the time of this review, studies have yet to find a significant effect of α1-antitrypsin augmentation on improving lung function, reducing exacerbation frequency, or on morbidity or mortality in patients with other chronic lung diseases. A recent study in patients with α1-antitrypsin deficiency suggested that this lack of clinical efficacy may be due to suboptimal dosing and that doubling the standard dose could further slow the loss of lung function [134]. Inhaled α1-antitrypsin therapy has also been evaluated in patients with CF. An initial open label study of inhaled α1-antitrypsin [135] showed that the therapy reduced NE abundance in BALF and that neutrophils added to BALF from post-treatment subjects were effective in killing *P. aeruginosa* compared to bacterial killing by neutrophils added to pretreatment BALF. However, although subsequent trials of inhaled α1-antitrypsin in CF have demonstrated that therapy is safe and tends to decrease NE in airway BALF or sputum, these studies have yet to show any improvement in lung function or in decreasing rates of exacerbations [136,137].

Other antineutrophil elastase therapies have been studied in clinical trials. Silvelestat is the only NE synthetic inhibitor approved for clinical use and is exclusively used in Japan and Korea to treat acute lung injury and respiratory distress syndrome. However, studies testing Silvelestat treatment for acute respiratory distress syndrome in the US were stopped early by the Data Safety Monitoring Board providing oversight for the study, due to increased long-term mortality for subjects on Silvelestat [138]. AZD9668 is a reversible and selective NE inhibitor that was tested for efficacy in COPD [139], CF [140], and bronchiectasis [121] in randomized, double-blind, placebo-controlled trials. Although AZD9668 decreased sputum measures of inflammation, these trials did not demonstrate significant improvement in lung function or symptom scores for any of these three protease-dominant diseases. These studies of well-characterized and potent antiproteases which failed to demonstrate a robust impact on clinical outcomes, suggest that the strategy of focusing solely on anti-NE activity alone will not be sufficient to block the unremitting inflammatory milieu in the airways to change the trajectory of clinical outcomes. Instead, a new strategy employing combination therapy and/or multi-function drugs that have antiprotease and anti-inflammatory properties may be a more successful strategy.

Flavonoids are polyphenolic compounds derived from plants. They have been investigated over the past two decades for antiprotease and anti-inflammatory activity [141]. Several flavonoid glucuronide derivatives at 1 μM inhibited NE release by 30–50% and at 10 μM, decreased ROS release by 50–70% from activated neutrophils [142]. Several modified flavonoids also have anti-elastase activity, with IC50 in the micromolar range [141]. However, these compounds have not yet been tested in chronic lung diseases characterized by neutrophil predominant inflammation.

Polysulfated glycosaminoglycans (GAGs) are potent anti-elastase drugs with multiple anti-inflammatory properties [143], and the prototypical drug in this class, heparin, has a strong record of safety and efficacy when administered for other lung disease indications such as asthma, acute lung injury, and smoke inhalation in humans [143]. In one double-blind, placebo-controlled pilot study, inhalation of unfractionated heparin as a therapeutic for COPD resulted in improved lung function, underscoring the significant promise of GAG therapy for chronic lung diseases [144]. Polysulfated GAGs, including 2-O, 3-O desulfated heparin (ODSH) [145], a polysulfated hyaluronan (GM-1111) [146], and non-saccharide glycosaminoglycan mimetic (NSGM) (G32) [147], are potent anti-elastase drugs that have minimal anticoagulant activity and, therefore, may be advantageous for chronic inhalation. We have demonstrated by in silico modeling and Michealis–Menten kinetics that ODSH functions by an allosteric mechanism, binding to basic amino acid residues outside the NE catalytic domain [145] and competing with sputum DNA for access to that site. G32 also binds in part to the allosteric domain but also interacts with Histidine in the catalytic domain [147], so G32 has a dual mechanism of action. ODSH, GM-1111, and G32 inhibit NE in CF sputum supernatant treated with dornase alfa and hypertonic saline, the current mucolytic and mucokinetic therapies for the CF airway. GM-1111 has also been effective to resolve chronic allergic rhinosinusitis in a mouse model [148].

Importantly, polysulfated GAGs also have multifunction anti-inflammatory properties. ODSH inhibits histone acetyltransferase activity, blocking acetylation of HMGB1 and preventing NE-triggered release of HMGB1 by macrophages in vitro [149]. Heparin and ODSH inhibit NFκB activation, block L- and P-selectin binding, and interfere with HMGB1–receptor for advanced glycation end-products (RAGE) interactions and S100A9/calgranulin–RAGE interactions [150,151]. Polysulfated hyaluronan resolves allergy-mediated and LL-37-mediated rhinosinusitis in a mouse model, supporting a broader anti-inflammatory activity for this novel drug [148,152]. Future comprehensive studies to explore how GAGs can be developed and implemented to treat chronic inflammatory airway diseases are warranted.

## 12. Summary

NE is critical for the host immune response to infection, but NE is also a major instigating factor for inflammation and airway injury in chronic inflammatory lung diseases. Because of the pleiotropic impact of NE activity, it is unlikely that inhibition of NE activity alone will resolve or reverse chronic inflammatory lung diseases. Instead, we propose a strategy of targeting multiple proteases and signaling pathways activated by NE to successfully inhibit inflammation and facilitate airway repair. GAGs may provide a cornerstone therapy in this new therapeutic strategy.

## Figures and Tables

**Table 1:** Extracellular neutrophil elastase: mechanisms for pathogenesis of chronic lung disease.

Airway Remodeling	Pro-Inflammatory Effects	Impaired Innate Immunity
Upregulates airway mucins, MUC5AC, MUC4, and MUC1 [13,14,15,16]; Triggers mucin secretion [17]	Activates TLR4 and upregulates IL-8 [18]	Degrades transferrin, lactoferrin [19] and midkine [20]
Stimulates goblet cell metaplasia ^a^ [21]	Activates IL-1α, IL-33, IL-36α, IL-36γ [22]	Cleaves phosphatidyl serine receptor resulting in efferocytosis failure [23]
Inhibits ciliary motility and injures cilia [24]	Activates High Mobility Group Box 1 [HMGB1] release [25]	Cleaves opsonins- C3bi [26], IgG [27], SP-D [28] SP-A [29]; Cleaves opsonin receptors [26]
Degrades CFTR [30] and activates ENaC [31] to promote airway dehydration	Increases cellular oxidative stress by releasing heme-free iron for uptake [32]	Cleaves lymphocyte receptors CD2, CD4, CD8 [33]; Cleaves neutrophil CXCR1 receptor [34]
Increases epithelial apoptosis [35]	Upregulates ceramide in vivo which mediates airway inflammation ^a^ [36]	Impairs macrophage phagocytic function [37]
Transiently down-regulates ErbB2 and suppresses epithelial proliferation [38]	Activates other proteases meprin alpha [16], matrix metalloprotease (MMP)-2 [39], MMP-9 [40], calpain-2 [30], Cathepsin B [39]	Impairs neutrophil *E. coli and S. aureus* killing [22]
Promotes epithelial cell cycle arrest [41] and senescence [42]	Degrades TIMP1 [40] and SLPI [43]	Blocks dendritic cell maturation [44]
Degrades extracellular matrix ^b^ [45]	Triggers NETs [46] and exosome release [47]	Cleaves fibrin degradation products that are chemotactic for PMN [48]

All results reported in human epithelial cells except the following: a, mouse; b, dog.
